# Osmolality Selectively Offsets the Impact of Hyperthermia on Mouse Skeletal Muscle *in vitro*

**DOI:** 10.3389/fphys.2018.01496

**Published:** 2018-10-31

**Authors:** Orlando Laitano, Laila H. Sheikh, Alex J. Mattingly, Kevin O. Murray, Leonardo F. Ferreira, Thomas L. Clanton

**Affiliations:** ^1^Department of Applied Physiology and Kinesiology, University of Florida, Gainesville, FL, United States; ^2^Colegiado de Educação Física, Federal University of Vale do São Francisco, Petrolina, Brazil

**Keywords:** heat stress, dehydration, oxidative stress, osmotic stress, rhabdomyolysis

## Abstract

Hyperthermia and dehydration can occur during exercise in hot environments. Nevertheless, whether elevations in extracellular osmolality contributes to the increased skeletal muscle tension, sarcolemmal injury, and oxidative stress reported in warm climates remains unknown. We simulated osmotic and heat stress, *in vitro*, in mouse limb muscles with different fiber compositions. Extensor digitorum longus (EDL) and soleus (SOL) were dissected from 36 male C57BL6J and mounted at optimal length in tissue baths containing oxygenated buffer. Muscles were stimulated with non-fatiguing twitches for 30 min. Four experimental conditions were tested: isotonic-normothermia (285 mOsm•kg^-1^ and 35°C), hypertonic-normothermia (300 mOsm•kg^-1^ and 35°C), isotonic-hyperthermia (285 mOsm•kg^-1^ and 41°C), and hypertonic-hyperthermia (300 mOsm•kg^-1^ and 41°C). Passive tension was recorded continuously. The integrity of the sarcolemma was determined using a cell-impermeable fluorescent dye and immunoblots were used for detection of protein carbonyls. In EDL muscles, isotonic and hypertonic-hyperthermia increased resting tension (*P* < 0.001). Whereas isotonic-hyperthermia increased sarcolemmal injury in EDL (*P* < 0.001), this effect was absent in hypertonic-hyperthermia. Similarly, isotonic-hyperthermia elevated protein carbonyls *(P* = 0.018), a response not observed with hypertonic-hyperthermia. In SOL muscles, isotonic-hyperthermia also increases resting tension (*P* < 0.001); however, these effects were eliminated in hypertonic-hyperthermia. Unlike EDL, there were no effects of hyperthermia and/or hyperosmolality on sarcolemmal injury or protein carbonyls. Osmolality selectively modifies skeletal muscle response to hyperthermia in this model. Fast-glycolytic muscle appears particularly vulnerable to isotonic-hyperthermia, resulting in elevated muscle tension, sarcolemmal injury and protein oxidation; whereas slow-oxidative muscle exhibits increased tension but no injury or protein oxidation under the conditions and duration tested.

## Introduction

Hyperthermia is a common feature of exercise in hot environments and is accompanied by sweat-induced dehydration when fluid losses are not adequately matched by fluid replacement. As sweat is normally hypotonic in relation to plasma, sweat losses are associated with a hypertonic plasma (Wendt et al., 2007). Several secondary factors related to both hyperthermia and dehydration may contribute to muscle injury in the heat. For instance, hyperthermia *per se* can induce increases in passive tension (Oliver et al., 2008), a response often attributed to elevations in intracellular calcium (Ca^2+^) release from the sarcoplasmic reticulum (SR) (Hill, 1972; van der Poel and Stephenson, 2002, 2007). Increases in extracellular osmolality associated with dehydration can also induce increases in muscle tension (Clausen et al., 1979; Farhat et al., 2018) by affecting muscle lattice structure and Ca^2+^ release (Clausen et al., 1979; Millman, 1998; Cleary et al., 2005). Calcium can influence cross bridge alignment, optimum myosin-actin interactions, and presumably susceptibility to stretch-induced damage leading to sarcolemmal injury. Extensive sarcolemmal injury is an underlying feature of exertional rhabdomyolysis, which is of concern in athletes as well as in active duty military personnel (Clarkson, 2006; Cleary et al., 2011; Rawson et al., 2017). The incidence of rhabdomyolysis is 5 to 7 times greater during summer months (Armed Forces Health Surveillance Bureau, 2017), suggesting that elevated temperatures and disorders of fluid balance may be contributing factors. However, the extent of osmotic and heat stress involvement and the mechanisms behind their influences are not well understood.

Hyperthermia stimulates the production of skeletal muscle reactive oxygen species (ROS) (Zuo et al., 2000). In humans, exercise-induced ROS are attenuated when elevations in body temperature are suppressed (Laitano et al., 2010; Hillman et al., 2011; Quindry et al., 2013; King et al., 2016). The production of ROS could contribute to injury of the sarcolemmal membrane, cytoskeleton and DNA (Powers and Jackson, 2008; Reid, 2016). ROS have been reported to contribute to SR-mediated Ca^2+^ release in this setting (van der Poel and Stephenson, 2007; Oliver et al., 2008), which can result in secondary mechanisms of injury through activation of proteases and phospholipases. The molecular sources of ROS produced during hyperthermia remain poorly understood, but ROS may be a result of skeletal muscle requirements for adapting to osmotic challenges (King et al., 2016), direct effects of hyperthermia (Zuo et al., 2000), or dehydration (Eisner et al., 2006; Laitano et al., 2012; King et al., 2016), or the heat-induced activation of eicosanoid pathways (Calderwood et al., 1989; Zuo et al., 2004). Carbonyl groups are produced on protein side chains when they are oxidized by ROS. Due to their high chemical stability, protein carbonyls have been used as biomarkers of oxidative stress (Dalle-Donne et al., 2003), including in exercise-hyperthermia settings (Souza-Silva et al., 2016), which makes them an attractive biomarker of protein oxidation in skeletal muscle *ex vivo* preparations.

In this study we tested the hypothesis that elevations in extracellular osmolality and temperature, within physiological ranges experienced by skeletal muscle during exercise in the heat (González-Alonso et al., 1997; Parkin et al., 1999; Laitano et al., 2012), contribute to changes in skeletal muscle resting tension. Secondary objectives were to evaluate differences in susceptibility to sarcolemmal injury between fast and slow-fiber dominant muscles and to determine whether protein oxidation was affected by these stressors.

## Materials and Methods

### Mice Care and Handling

The study protocol was approved by the University of Florida Institutional Animal Care and Use Committee. Thirty-six male adult C57BL/6J mice (22–26 g) were obtained from Jackson Laboratories (Bar Harbor, ME, United States) and housed at the University of Florida on a 12/12 h light-dark cycle with the dark cycle between 07:00 PM and 07:00 AM. The temperature and humidity of the room was maintained at 19–23°C and approximately 40–60%, respectively. Animals were brought from the animal care facility to the laboratory one-day prior to the experiment in their own cages, remaining on a 12/12 light cycle, in order to adapt to the laboratory and to recover from the stress of transport.

### Skeletal Muscle Excision and Resting Tension

Prior to amputation of both legs, mice were anesthetized with 3% isoflurane and skin was removed with forceps. Mice were euthanized immediately after the amputation. Legs were immediately transferred to a dish containing freshly prepared, modified isotonic Krebs-Henseleit (KH) buffer described in detail in Table 1. The buffer was continuously bubbled with 95%O_2_/5%CO_2_. Soleus (SOL) of the right leg and extensor digitorum longus (EDL) of the left leg were quickly removed under a magnifying microscope and mounted in water-jacketed tissue baths containing 4 ml of either isotonic (285 mOsm•kg^-1^) or hypertonic (300 mOsm•kg^-1^) KH buffer with 95%O_2_ and 5%CO_2_. We used 4-0 silk suture to tie the proximal tendon to the force transducer (GrassFT03). The distal tendon was attached to a glass rod using a loop of a suture. Initially, muscles were kept resting in the baths at optimal length for 10 min period under isotonic buffer and 35°C temperature. Thereafter, we switched the connectors of the water circulation unit to 41°C for hyperthermia conditions or simply maintained at 35°C for control conditions and we switched the buffer to fresh hypertonic or isotonic depending on the condition studied. For hyperthermia experiments, the buffer added to the bath at the time of temperature increase (after 10 min of resting equilibration) was pre heated to 42°C to avoid delays required to achieve the desired experimental temperature. Buffer osmolality was manipulated by increasing sodium chloride (NaCl) content of the buffer as described in Table 1. Buffer osmolality was confirmed using a vapor pressure micro osmometer (Vapro 5520, Wescor Biomedical Systems). EDL and SOL muscles were chosen because they are known to express a more pronounced glycolytic and oxidative phenotypes, respectively (Augusto et al., 2004).

**Table 1:** Composition of the modified Krebs-Henseleit (KH) buffers used in the experiments.

	Isotonic (mM)	Hypertonic (mM)	Molar Mass (g/mol)
Sodium sulfate (Na_2_SO_4_)	0.45	0.45	142.04
Disodium phosphate (Na_2_HPO_4_)	0.6	0.6	141.96
Magnesium chloride (MgCl_2_)	1.0	1.0	95.2
Potassium chloride (KCl)	5.9	5.9	74.55
Calcium chloride (CaCl_2_)	2.0	2.0	110.98
Sodium bicarbonate (NaHCO_3_)	21.0	21.0	84.01
Sodium chloride (NaCl)	121.0	127.3	58.44
Glucose (C_6_H_12_O_6_)	11.5	11.5	180
Tubocurarine chloride (C_37_H_42_Cl_2_N_2_O_6_)	0.01	0.01	681.7

*Osmolality was 285 mOsm•kg^-1^ for isotonic and 300 mOsm•kg^-1^ for hypertonic. Hypertonicity was achieved by increasing sodium chloride content in the buffer.*

Muscles were electrically stimulated to perform 30 min of non-fatiguing twitch contractions at a frequency of 5 contractions/min (Grass S88 Square Pulse Stimulator), while maintained at initial length. Resting tension was recorded (DasyLab Software) throughout the twitch contraction protocol by having the muscles attached to a strain gauge force transducer. After the stimulation protocol, muscles were exposed to 0.15% wt/v of procion orange MX2R (Sigma Aldrich, Ref. 311391) in isotonic KH buffer with 95% O_2_/5% CO_2_ at 35°C for 45 min, followed by a 15-min rinse with fresh buffer. Muscles were immediately embedded in a medium for frozen tissue specimens to ensure optimal cutting temperature (OCT, Sakura Tissue Tek) and frozen with liquid nitrogen in cold 2-methylbutane.

### Skeletal Muscle Histology and Microscopy Assessments

Soleus (SOL) and EDL samples were cut in sections ranging from 10–30 μm by using a cryostat (Leica Biosystems, model CM3050 S) set at -20°C. Slices were placed on glass slides and were washed with phosphate saline buffer to remove OCT. We used an anti-fade mounting medium (Vectashield – H-1000) to prevent photobleaching. Slides were kept at 4°C until microscopy analysis. Multiple images of each section were recorded with an epifluorescence microscope (Olympus DP71) using standard filter settings for fluorescein. The numbers of cells containing procion orange MX2R were delineated as the percentage of total number of cells in the whole field and were analyzed by two independent, trained raters who were blinded to the experimental conditions.

### SDS-PAGE and Western Blot

Skeletal muscle samples were washed in phosphate saline buffer and subsequently homogenized using a Kontes Duall Homogenizer in cell lysis buffer (No. 9803; Cell Signaling Technology) containing 20 mM Tris–HCl (pH 7.5), 150 mM NaCl, 1 mM Na_2_EDTA, 1 mM EGTA, 1% Triton-X, 2.5 mM sodium pyrophosphate, 1 mM-glycerophosphate 1 mM Na_3_PO_4_, 1 μg/ml leupeptin, and protease and phosphatase inhibitor cocktail (no. 5872; Cell Signaling Technology). We performed a detergent compatible assay (DC Assay, Bio-Rad Laboratories) to determine the total protein content of the lysates. The lysates were mixed 1:1 with 2X Laemmli sample buffer (Bio-Rad Laboratories). We loaded 20 μg/lane into 4–20% stain-free TGX gels (Bio-Rad Laboratories) and performed electrophoresis at 200 V for 50 min with the chamber buried in ice. Proteins were transferred to a nitrocellulose membrane at 100 mA for 18 h at 4°C. The membranes were scanned with Revert Total Protein Stain (Li-COR, Lincoln, NE, United States) at the 700 nm channel to determine the total amount of protein transferred. We then washed the membranes to remove the revert staining, according to manufacturer instructions, and blocked the membrane immediately after by using Li-COR PBS Blocking Buffer (Li-COR, Lincoln, NE, United States) for 1 h at room temperature.

To determine protein carbonyls, we used the Oxi-Select Protein Carbonyl Immunoblot kit (STa-308, Cell Biolabs) following the manufacturer recommendations. In brief, to promote protein derivatization, we incubated the membranes with TBS + 20% methanol for 5 min and then washed in 2N HCL for 5 min. We then incubated the membranes in 2,4-Dinitrophenylhydrazine (DNPH) for 5 min in the dark. Thereafter, the membranes were washed 3X in 2N HCL and 5× in 50% methanol, each wash was for 5 min. Membranes were further incubated with anti-DNPH primary antibody diluted 1:100 in Li-COR PBS Blocking Buffer for 1 h at room temperature. After 3 washes with TBS-T, the membranes were finally incubated with anti-rabbit secondary antibody diluted 1:20000 in Li-COR PBS Blocking Buffer for another hour. We scanned the membranes using an Odyssey Infrared Imaging system (LI-COR CLX) at 800 nm channel. The immunoblot signal was normalized to the total protein signal measured in the corresponding membrane lanes. These procedures are consistent with recent recommendations for data analysis of Western Blots using fluorescence methods (Eaton et al., 2013; Murphy and Lamb, 2013; Bass et al., 2017).

### Statistical Analysis

Data are reported as mean ± standard deviation, unless otherwise stated. Data distribution was determined by using the Shapiro–Wilk test. We used repeated measures two-way ANOVA to compare data over time and independent two-way ANOVA to compare means among treatments. Tukey’s *post hoc* was used to confirm where the differences occurred over time and among groups. To test the hypothesis, we predetermined the 95% level of confidence as the minimum criterion to denote a statistical difference (*p* ≤ 0.05). All analyses were made using SAS JMP Pro v.12.0 and GraphPad Prism v. 7.0 software.

## Results

**FIGURE 1:**
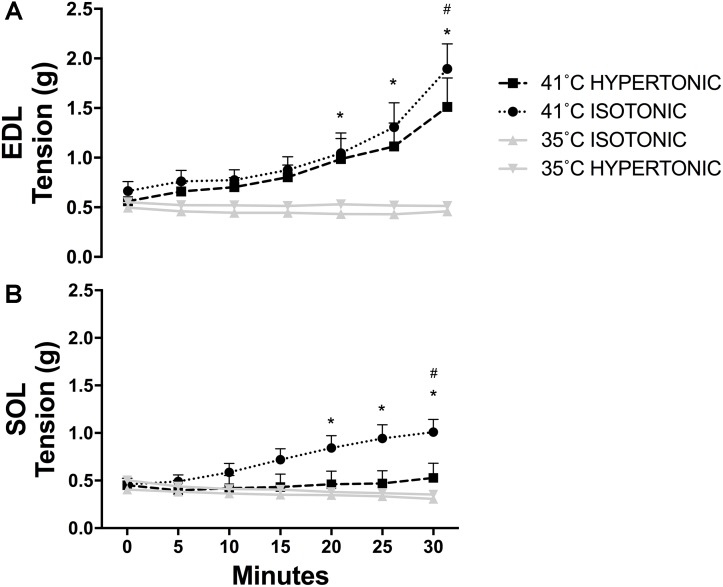
Skeletal muscle resting tension recorded throughout twitch contractions for EDL **(A)** ^∗^*P* < 0.05 over time, ^#^*P* < 0.05 in comparison to 35°C isotonic and hypertonic; and SOL **(B)** ^∗^*P* < 0.05 over time, ^#^*P* < 0.05 in comparison to 35°C isotonic, hypertonic, and 41°C hypertonic. *N* = 8 for 35°C and *N* = 10 for 41°C conditions per group. Repeated measures two-way ANOVA was used over time and independent two-way ANOVA was used to compare different experimental conditions.

Isotonic hyperthermia (41°C) increased resting tension in EDL [*F*(6,192) = 22.24, *P* < 0.001] over time. There was an increase in tension from baseline starting at 20 min (0 min vs. 20 min, *P* = 0.007) after the onset of hyperthermia. The tension remained elevated at 25 min (0 min vs. 25 min, *P* < 0.001) and 30 min (0 vs. 30 min, *P* < 0.001). The highest tension achieved by EDL muscles at 30 min with isotonic hyperthermia was higher than the tension achieved with isotonic (*P* = 0.008) and hypertonic normothermia (*P* = 0.023) as shown in Figure 1A. Similarly, hypertonic hyperthermia also increased tension over time in EDL. The tension increased at 20 min (*P* = 0.001) and remained elevated at 25 min (*P* < 0.001) and 30 min (*P* < 0.001). The highest tension achieved at 30 min with hypertonic hyperthermia was higher than the tension recorded for muscles under isotonic (*P* < 0.01) and hypertonic normothermia (*P* = 0.01) at the same time point.

In SOL muscles, only isotonic hyperthermia caused an increase in tension over time [*F*(6,186) = 3.73, *P* < 0.001]. Resting tension began to increase at 20 min (0 min vs. 20 min, *P* < 0.001) and remained elevated at 25 min (0 min vs. 25 min, *P* < 0.001) and 30 min (0 min vs. 30 min, *P* < 0.001). Interestingly, there was no increase in resting tension with hypertonic hyperthermia. The tension observed for SOL at 30 min with isotonic hyperthermia was higher than the tension recorded with both isotonic (*P* = 0.001) and hypertonic normothermia (*P* = 0.003) and higher than the tension recorded at 30 min with hypertonic hyperthermia (*P* = 0.022) as can be seen in Figure 1B.

**FIGURE 2:**
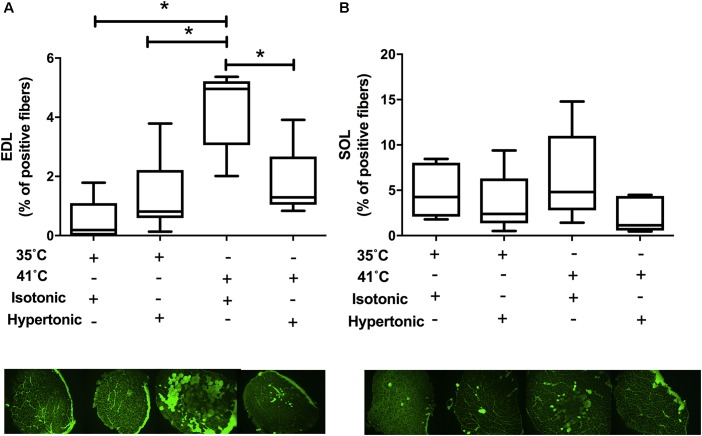
Procion orange leakage into skeletal muscle fibers under isotonic and hypertonic conditions with and without heat stress (*N* = 8 for 35°C and *N* = 10 for 41°C conditions). Pictures are from representative muscles at respective experimental condition. EDL muscle results are displayed in panel **A** and SOL data in panel **B**. Independent two-way ANOVA was used to compare different experimental conditions. Data are median of percentage with interquartile range. ^∗^*P* < 0.05.

Isotonic hyperthermia caused sarcolemmal injury in EDL muscles as assessed by the presence of cell-impermeable dye in individual muscle fibers [*F*(3,18) = 10.42, *P* < 0.001]. There was an increase in the percentage of fluorescent dye-positive fibers with isotonic hyperthermia in comparison to both isotonic (*P* < 0.001) and hypertonic normothermia (*P* = 0.002). Interestingly, isotonic hyperthermia showed a higher percentage of dye-positive fibers than hypertonic hyperthermia (*P* = 0.013), as seen in Figure 2A. There was no effect of any experimental treatment on SOL muscle permeability, which suggests a greater tolerance of SOL muscle to sarcolemma injury induced by hyperthermia and changes in osmolality, Figure 2B.

**FIGURE 3:**
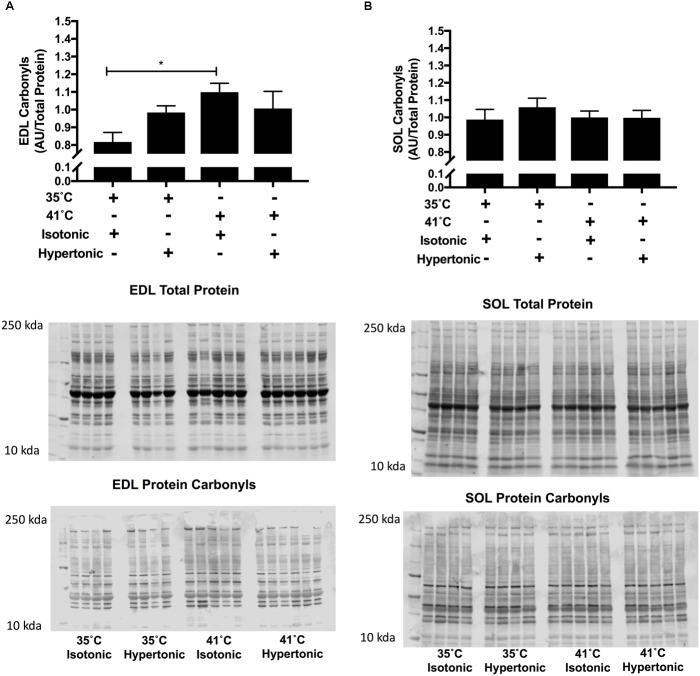
Protein carbonyls for EDL **(A)** and SOL **(B)** immunoblot relative to total protein (*N* = 8 for 35°C and *N* = 10 for 41°C conditions, ^∗^*P* < 0.05). Independent two-way ANOVA was used to compare different experimental conditions. Data are arbitrary units from fluorescent signal intensity divided by total protein.

Isotonic hyperthermia increased protein carbonyls in EDL muscles *F*(3,18) = 3.87, *P* = 0.018. While there was an increase in protein carbonyls with isotonic hyperthermia in comparison to isotonic normothermia (*p* = 0.009), no differences were observed with hypertonic normothermia or hyperthermia as seen in Figure 3, panel A. In SOL muscles, hyperthermia had no effects on protein carbonyls regardless of the osmolality employed.

## Discussion

The present study demonstrates that elevations in temperature, typical of skeletal muscle temperatures during exercise in the heat (Parkin et al., 1999), resulted in significant elevations in passive tension in both fast glycolytic and slow oxidative skeletal muscles. Our main finding was that the elevation in passive force was prevented in slow oxidative muscles when hyperthermia was accompanied by increased extracellular osmolality. In fast glycolytic muscle, there was evidence for both sarcolemmal injury and increased protein carbonyls that were not seen in slow oxidative muscle with hyperthermia. Additionally, hypertonic hyperthermia attenuated sarcolemmal injury and elevations in protein carbonyls in fast glycolytic muscle. In neither skeletal muscle type, did extracellular osmolality alone cause increase in tension, sarcolemmal injury or increases in protein carbonyls.

Our findings of increased skeletal muscle resting tension with hyperthermia are consistent with previous studies exploring the impact of temperature on skeletal muscle physiology (Hill, 1972; Oliver et al., 2008). There is consensus that the changes in skeletal muscle resting tension with hyperthermia involve altered Ca^2+^ homeostasis (van der Poel and Stephenson, 2002, 2007). These changes in Ca^2+^ homeostasis with hyperthermia are triggered, at least in part, by changes in fiber volume as hyperthermia is thought to participate in skeletal muscle fiber swelling responses (Usher-Smith et al., 2009; King et al., 2016). Swelling can increase cation permeability through the sarcolemmal membrane, which leads to extracellular Ca^2+^ influx into skeletal muscle fibers (King et al., 2016). Hyperthermia has also been shown to increase intracellular Ca^2+^ leakage from the SR in rodents (van der Poel and Stephenson, 2007). Whether the increased resting muscle tension caused by hyperthermia arises from the impact of elevated Ca^2+^ on the contractile (i.e., actin-myosin cross bridges) or elastic (i.e., titin) elements of the skeletal muscle remains unknown and warrants further experimentation. Titin is a potential candidate due to its known sensitivity to Ca^2+^ (Labeit et al., 2003) and its participation in force transmission at the Z line and resting tension in the I band region of striated muscles (Wang et al., 1991; Itoh-Satoh et al., 2002).

This is the first study to demonstrate that elevations in extracellular osmolality offset the impact of hyperthermia, even though the specific responses appear to be unique to fiber phenotype (i.e., slow oxidative vs. fast glycolytic). The increase in extracellular osmolality could counteract the impact in hyperthermia-induced skeletal muscle fiber swelling by activation of regulatory volume response pathways in slow oxidative skeletal muscle fibers (Usher-Smith et al., 2009; King et al., 2016), whereas fast glycolytic fibers may have less sensitivity or lower abundance of proteins involved in this regulatory volume response. This resonates with many previous investigations that have reported greater sensitivity to most forms of injury in fast twitch muscle, as reviewed in (Tiidus, 2008). Nevertheless, follow-up studies employing a wider range of osmolality and temperature are required to demonstrate when this interaction starts to display protective or dysfunctional features in fast-glycolytic skeletal muscle fibers.

Isotonic hyperthermia induced sarcolemmal injury in fast glycolytic muscles and this response was attenuated by hypertonic hyperthermia. Our data for sarcolemmal injury, using a cell-impermeable dye, suggests that fast-twitch muscles are more susceptible to heat induced-injury, but the degree of injury is attenuated when osmolality is elevated. Slow-twitch muscle fibers did not display significant injury with hyperthermia, which might indicate a more resistant phenotype to this stressor (Tiidus, 2008). In alignment with our present findings, a recent study in rodents also demonstrated greater sensitivity to osmolality changes induced by severe water deprivation in fast glycolytic than in slow oxidative muscles (Farhat et al., 2018).

We observed an increase in hyperthermia-induced skeletal muscle protein carbonyls in fast-twitch muscles. This is consistent with previous findings demonstrating that heat stress increases ROS formation in animal and human models. For example, rat diaphragm generates elevated intracellular and extracellular superoxide within a few minutes of exposure to 42°C (Zuo et al., 2000). Mouse EDL fibers exposed to 40°C demonstrate a leaky SR Ca^2+^ pump attributed to the effects of ROS formation (van der Poel and Stephenson, 2007). Besides causing an increase in tension, elevations in intracellular Ca^2+^ concentration can activate proteases and phospholipases that are known to increase ROS production and induce further injury (Powers and Jackson, 2008). Hyperthermia, with and without dehydration, exacerbates markers of ROS production in the circulation of resting and exercising humans (Laitano et al., 2010, 2012). In the present study, we observed that the increase in protein carbonyls was halted when hyperthermia was superimposed with extracellular hyperosmolality. These results suggest that physiological elevations in extracellular osmolality might be necessary to counteract the potentially detrimental effects of hyperthermia (e.g., augmented protein oxidation) in skeletal muscles of predominantly glycolytic fiber type compositions.

The source of hyperthermia-induced ROS that cause protein oxidation in skeletal muscle remains a matter of debate. A potential source of ROS in skeletal muscle during heat/osmotic disturbances are the NAD(P)H oxidases enzyme family (Ferreira and Laitano, 2016). Although osmotic disturbances have been associated with increased NAD(P)H oxidase activity in skeletal muscle (Martins et al., 2008), a causal role of hyperthermia upon this enzyme family remains to be established. A possible explanation for the different protein carbonyls response observed between fast and slow muscles in the present study could be that slow oxidative muscles contain a much greater abundance of antioxidant defense mechanisms compared to fast glycolytic muscles (Lawler et al., 1993) and even though the same underlying cell volume homeostatic pathways may be active, they may not result in significant protein oxidation or membrane injury, but this statement requires further investigation.

Our *in vitro* preparation was designed to mimic physiological alterations observed during an exercise session in the heat with moderate sweat-induced dehydration (e.g., within 4% reductions in body mass). Studies have reported increases in skeletal muscle temperature from 36°C to 41°C (Parkin et al., 1999) and in blood osmolality from ∼280 to 300 mOsm•kg^-1^ in humans exercising in the heat and eliciting 4% dehydration (González-Alonso et al., 1997; Laitano et al., 2012). Therefore, our *in vitro* preparation was consistent with hyperthermia and hyperosmolality observed in moderately dehydrated humans exercising in the heat. Severe heat injury is generally associated with accompanying effects of intense muscle contractions, particularly eccentric contractions. We did not incorporate maximal contractions into this experiment in order to isolate the effects of heat and osmolality. We conjecture that the subtler changes we observe here, set muscles up for much more intense injury during eccentric contractions associated with exercise. Recent experiments in humans combining mild heat exposure preceding eccentric contractions have shown no influence of pre-heating to 40°C on the extent of injury (Castellani et al., 2016). However, limitations inherent to the penetration, duration, and intensity of the heat stimulus make it still an open question and warrants further investigation.

Among the disadvantages of skeletal muscles *in vitro* preparations is the excessive PO_2_ exposure to peripheral fibers and the diminished O_2_ perfusion in the fibers located at the core. In this model, O_2_ diffusion is the transport method of O_2_ delivery and while more peripheral fibers are adequately, or even excessively, exposed to O_2_, fibers located in the core of the muscle bundle can experience lower PO_2_, presumably leading to hypoxia. This is a concern in preparations using hyperthermia since skeletal muscle oxygen consumption is increased by heat and because the PO_2_ in the bath is constant, PO_2_ is decreased in the core of the muscle. This has been debated before and while preparations using rat muscles demonstrate a hypoxic core in sections, mice skeletal muscles are small enough to allow adequate O_2_ diffusion from the bath to the fibers, particularly when in a near resting-state. To address this concern we have employed the equation proposed by Barclay (2005) where the distance from the surface of the muscle to the radial location where PO_2_ is 0 for different skeletal muscle types. In both EDL and SOL muscles this distance is negligible within the temperature range used in the present study.

Since the responses of hyperosmolality on hyperthermia-induced skeletal muscle alterations were largely unknown, the set of experiments performed were not designed to define the conclusive mechanisms behind the apparent attenuation caused by hyperosmolality on heat-induced increases in resting tension in slow-twitch muscles nor to explain the apparent protective feature of hyperosmolality against oxidative stress and sarcolemmal damage in fast glycolytic fibers. On the other hand, the present results set the ground for future studies to explore the mechanisms behind the responses herein described as well as to establish whether these findings can be extended to human skeletal muscle physiology. For instance, at a more molecular level, whether Ca^2+^ homeostasis is indeed the reason why hyperosmolality attenuates the rise in tension seen in skeletal muscle warrants further investigation. Likewise, the source of oxidants associated with heat-induced protein oxidation and fiber type specificity, whether it comes primarily from mitochondrial sources or changes in NADPH oxidase activity remains to be explored.

Can our results have implications for human performance and susceptibility to muscle injury during exercise in hot environments? It is difficult to translate directly to *in vivo* human conditions because it is challenging to predict precisely what the osmolality would be in the interstitial space surrounding an actively contracting muscle, due to the continual flux of K^+^ and other solutes. Also, we manipulated osmolality by increasing NaCl content in the extracellular buffer while all other solutes were kept under the same concentration as in isotonic buffer, this differs from increases in extracellular osmolality seen in hypohydration states where usually all solutes increase somewhat evenly with fluid losses. Our results highlight and support further precautions against the consequences of overhydration during exercise in the heat, beyond the usual discussion of electrolyte imbalances. We have proposed that muscle cell swelling during intense exercise in the heat may be a significant source of ROS formation and injury (King et al., 2016) and therefore, in addition to the suggestions of deleterious effects of hyponatremia on muscle injury (Cairns and Hew-Butler, 2016), there may be additional risks of muscle injury associated with cell swelling.

## Conclusion

In summary, we demonstrate for the first time that osmolality selectively modifies mouse skeletal muscle response to hyperthermia in this model. Specifically, superimposing hyperosmolality to hyperthermia attenuates resting tension in slow twitch skeletal muscle and prevents sarcolemma injury and protein oxidation in fast twitch muscles. Our results are consistent with slow twitch muscles being more resistant to hyperthermia than fast twitch ones regarding protein oxidation and sarcolemma injury.

## Author Contributions

OL, LF, and TC conceived the study. OL, LS, AM, and KM performed muscle dissections and *ex vivo* experiments. OL wrote the manuscript. All authors edited and approved the final version of the manuscript.

## Conflict of Interest Statement

The authors declare that the research was conducted in the absence of any commercial or financial relationships that could be construed as a potential conflict of interest.

## References

[B1] Armed Forces Health Surveillance Bureau. (2017). Update: exertional rhabdomyolysis, active component, U.S. Armed Forces, 2012-2016. *MSMR* 24 14–18. 28358521

[B2] Augusto V. Padovani C. R. Campos G. E. R. (2004). Skeletal muscule fiber types in C57BL6J mice. *Braz. J. Morphol. Sci.* 21 89–94.

[B3] Barclay C. J. (2005). Modelling diffusive O^2^ supply to isolated preparations of mammalian skeletal and cardiac muscle. *J. Muscle Res. Cell Motil.* 26 225–235. 10.1007/s10974-005-9013-x 16322911

[B4] Bass J. J. Wilkinson D. J. Rankin D. Phillips B. E. Szewczyk N. J. Smith K. (2017). An overview of technical considerations for Western blotting applications to physiological research. *Scand. J. Med. Sci. Sports* 27 4–25. 10.1111/sms.12702 27263489 PMC5138151

[B5] Cairns R. S. Hew-Butler T. (2016). Proof of concept: hypovolemic hyponatremia may precede and augment creatine kinase elevations during an ultramarathon. *Eur. J. Appl. Physiol.* 116 647–655. 10.1007/s00421-015-3324-4 26747653

[B6] Calderwood S. K. Bornstein B. Farnum E. K. Stevenson M. A. (1989). Heat shock stimulates the release of arachidonic acid and the synthesis of prostaglandins and leukotriene B4 in mammalian cells. *J. Cell. Physiol.* 141 325–333. 10.1002/jcp.1041410214 2553753

[B7] Castellani J. W. Zambraski E. J. Sawka M. N. Urso M. L. (2016). Does high muscle temperature accentuate skeletal muscle injury from eccentric exercise? *Physiol. Rep.* 4:e12777. 10.14814/phy2.12777 27185904 PMC4873630

[B8] Clarkson P. M. (2006). Case report of exertional rhabdomyolysis in a 12-year-old boy. *Med. Sci. Sports Exerc.* 38 197–200. 10.1249/01.mss.0000183478.12106.04 16531884

[B9] Clausen T. Dahl-Hansen A. B. Elbrink J. (1979). The effect of hyperosmolarity and insulin on resting tension and calcium fluxes in rat soleus muscle. *J. Physiol.* 292 505–526. 490385 10.1113/jphysiol.1979.sp012868 PMC1280875

[B10] Cleary M. A. Sadowski K. A. Lee S. Y.-C. Miller G. L. Nichols A. W. (2011). Exertional rhabdomyolysis in an adolescent athlete during preseason conditioning: a perfect storm. *J. Strength Cond. Res.* 25 3506–3513. 10.1519/JSC.0b013e318216302f 22080315

[B11] Cleary M. A. Sweeney L. A. Kendrick Z. V. Sitler M. R. (2005). Dehydration and symptoms of delayed-onset muscle soreness in hyperthermic males. *J. Athl. Train.* 40 288–297. 16404450 PMC1323290

[B12] Dalle-Donne I. Rossi R. Giustarini D. Milzani A. Colombo R. (2003). Protein carbonyl groups as biomarkers of oxidative stress. *Clin. Chim. Acta* 329 23–38. 10.1016/S0009-8981(03)00003-2 12589963

[B13] Eaton S. L. Roche S. L. Hurtado M. L. Oldknow K. J. Farquharson C. Gillingwater T. H. (2013). Total protein analysis as a reliable loading control for quantitative fluorescent western blotting. *PLoS One* 8:e72457. 10.1371/journal.pone.0072457 24023619 PMC3758299

[B14] Eisner V. Criollo A. Quiroga C. Olea-Azar C. Santibañez J. F. Troncoso R. (2006). Hyperosmotic stress-dependent NFkappaB activation is regulated by reactive oxygen species and IGF-1 in cultured cardiomyocytes. *FEBS Lett.* 580 4495–4500. 10.1016/j.febslet.2006.07.029 16870182

[B15] Farhat F. Grosset J. F. Canon F. (2018). Water deprivation decreases strength in fast twitch muscle in contrast to slow twitch muscle in rat. *Acta Physiol.* 224:e13072. 10.1111/apha.13072 29633518

[B16] Ferreira L. F. Laitano O. (2016). Regulation of NADPH oxidases in skeletal muscle. *Free Radic. Biol. Med.* 98 18–28. 10.1016/j.freeradbiomed.2016.05.011 27184955 PMC4975970

[B17] González-Alonso J. Mora-Rodríguez R. Below P. R. Coyle E. F. (1997). Dehydration markedly impairs cardiovascular function in hyperthermic endurance athletes during exercise. *J. Appl. Physiol.* 82 1229–1236. 10.1152/jappl.1997.82.4.1229 9104860

[B18] Hill D. K. (1972). Resting tension and the form of the twitch of rat skeletal muscle at low temperature. *J. Physiol.* 221 161–171. 10.1113/jphysiol.1972.sp009746 5016979 PMC1331327

[B19] Hillman A. R. Vince R. V. Taylor L. McNaughton L. Mitchell N. Siegler J. (2011). Exercise-induced dehydration with and without environmental heat stress results in increased oxidative stress. *Appl. Physiol. Nutr. Metab.* 36 698–706. 10.1139/h11-080 21980993

[B20] Itoh-Satoh M. Hayashi T. Nishi H. Koga Y. Arimura T. Koyanagi T. (2002). Titin mutations as the molecular basis for dilated cardiomyopathy. *Biochem. Biophys. Res. Commun.* 291 385–393. 10.1006/bbrc.2002.6448 11846417

[B21] King M. A. Clanton T. L. Laitano O. (2016). Hyperthermia, dehydration, and osmotic stress: unconventional sources of exercise-induced reactive oxygen species. *Am. J. Physiol. Regul. Integr. Comp. Physiol.* 310 R105–R114. 10.1152/ajpregu.00395.2015 26561649

[B22] Labeit D. Watanabe K. Witt C. Fujita H. Wu Y. Lahmers S. (2003). Calcium-dependent molecular spring elements in the giant protein titin. *Proc. Natl. Acad. Sci.* 100 13716–13721. 10.1073/pnas.2235652100 14593205 PMC263879

[B23] Laitano O. Kalsi K. K. Pearson J. Lotlikar M. Reischak-Oliveira A. González-Alonso J. (2012). Effects of graded exercise-induced dehydration and rehydration on circulatory markers of oxidative stress across the resting and exercising human leg. *Eur. J. Appl. Physiol.* 112 1937–1944. 10.1007/s00421-011-2170-2 21932069

[B24] Laitano O. Kalsi K. K. Pook M. Oliveira A. R. González-Alonso J. (2010). Separate and combined effects of heat stress and exercise on circulatory markers of oxidative stress in euhydrated humans. *Eur. J. Appl. Physiol.* 110 953–960. 10.1007/s00421-010-1577-5 20658249

[B25] Lawler J. M. Powers S. K. Visser T. Van Dijk H. Kordus M. J. Ji L. L. (1993). Acute exercise and skeletal muscle antioxidant and metabolic enzymes: effects of fiber type and age. *Am. J. Physiol.* 265 R1344–R1350. 10.1152/ajpregu.1993.265.6.R1344 8285276

[B26] Martins A. S. Shkryl V. M. Nowycky M. C. Shirokova N. (2008). Reactive oxygen species contribute to Ca2 + signals produced by osmotic stress in mouse skeletal muscle fibres. *J. Physiol.* 586 197–210. 10.1113/jphysiol.2007.146571 17974587 PMC2375568

[B27] Millman B. M. (1998). The filament lattice of striated muscle. *Physiol. Rev.* 78 359–391. 10.1152/physrev.1998.78.2.359 9562033

[B28] Murphy R. M. Lamb G. D. (2013). Important considerations for protein analyses using antibody based techniques: down-sizing Western blotting up-sizes outcomes. *J. Physiol.* 591 5823–5831. 10.1113/jphysiol.2013.263251 24127618 PMC3872754

[B29] Oliver S. R. Wright V. P. Parinandi N. Clanton T. L. (2008). Thermal tolerance of contractile function in oxidative skeletal muscle: no protection by antioxidants and reduced tolerance with eicosanoid enzyme inhibition. *Am. J. Physiol. Regul. Integr. Comp. Physiol.* 295 R1695–R1705. 10.1152/ajpregu.90429.2008 18768765 PMC2584865

[B30] Parkin J. M. Carey M. F. Zhao S. Febbraio M. A. (1999). Effect of ambient temperature on human skeletal muscle metabolism during fatiguing submaximal exercise. *J. Appl. Physiol.* 86 902–908. 10.1152/jappl.1999.86.3.902 10066703

[B31] Powers S. K. Jackson M. J. (2008). Exercise-induced oxidative stress: cellular mechanisms and impact on muscle force production. *Physiol. Rev.* 88 1243–1276. 10.1152/physrev.00031.2007 18923182 PMC2909187

[B32] Quindry J. Miller L. McGinnis G. Kliszczewiscz B. Slivka D. Dumke C. (2013). Environmental temperature and exercise-induced blood oxidative stress. *Int. J. Sport Nutr. Exerc. Metab.* 23 128–136. 23532145 10.1123/ijsnem.23.2.128

[B33] Rawson E. S. Clarkson P. M. Tarnopolsky M. A. (2017). Perspectives on exertional rhabdomyolysis. *Sports Med.* 47 33–49. 10.1007/s40279-017-0689-z 28332112 PMC5371628

[B34] Reid M. B. (2016). Reactive oxygen species as agents of fatigue. *Med. Sci. Sports Exerc.* 48 2239–2246. 10.1249/MSS.0000000000001006 27285492

[B35] Souza-Silva A. A. Moreira E. de Melo-Marins D. Schöler C. M. de Bittencourt P. I. H. Laitano O. (2016). High intensity interval training in the heat enhances exercise-induced lipid peroxidation, but prevents protein oxidation in physically active men. *Temperature* 3 167–175. 10.1080/23328940.2015.1132101 27227083 PMC4861192

[B36] Tiidus P. M. (2008). *Skeletal Muscle Damage and Repair.* Champaign, IL: Human Kinetics.

[B37] Usher-Smith J. A. Huang C. L.-H. Fraser J. A. (2009). Control of cell volume in skeletal muscle. *Biol. Rev. Camb. Philos. Soc.* 84 143–159. 10.1111/j.1469-185X.2008.00066.x 19133959

[B38] van der Poel C. Stephenson D. G. (2002). Reversible changes in Ca^2+^ - activation properties of rat skeletal muscle exposed to elevated physiological temperatures. *J. Physiol.* 544 765–776. 12411522 10.1113/jphysiol.2002.024968 PMC2290629

[B39] van der Poel C. Stephenson D. G. (2007). Effects of elevated physiological temperatures on sarcoplasmic reticulum function in mechanically skinned muscle fibers of the rat. *Am. J. Physiol. Cell Physiol.* 293 C133–C141. 10.1152/ajpcell.00052.2007 17344316

[B40] Wang K. McCarter R. Wright J. Beverly J. Ramirez-Mitchell R. (1991). Regulation of skeletal muscle stiffness and elasticity by titin isoforms: a test of the segmental extension model of resting tension. *Proc. Natl. Acad. Sci.* 88 7101–7105. 10.1073/pnas.88.16.7101 1714586 PMC52241

[B41] Wendt D. van Loon L. J. C. Lichtenbelt W. D. van M. (2007). Thermoregulation during exercise in the heat: strategies for maintaining health and performance. *Sports Med.* 37 669–682. 17645370 10.2165/00007256-200737080-00002

[B42] Zuo L. Christofi F. L. Wright V. P. Bao S. Clanton T. L. (2004). Lipoxygenase-dependent superoxide release in skeletal muscle. *J. Appl. Physiol.* 97 661–668. 10.1152/japplphysiol.00096.2004 15107407

[B43] Zuo L. Christofi F. L. Wright V. P. Liu C. Y. Merola A. J. Berliner L. J. (2000). Intra- and extracellular measurement of reactive oxygen species produced during heat stress in diaphragm muscle. *Am. J. Physiol. Cell Physiol.* 279 C1058–C1066. 11003586 10.1152/ajpcell.2000.279.4.C1058

